# Game Theory-Based Cooperation for Underwater Acoustic Sensor Networks: Taxonomy, Review, Research Challenges and Directions

**DOI:** 10.3390/s18020425

**Published:** 2018-02-01

**Authors:** Dalhatu Muhammed, Mohammad Hossein Anisi, Mahdi Zareei, Cesar Vargas-Rosales, Anwar Khan

**Affiliations:** 1 Department of Computer System and Technology, Faculty of Computer Science and Information Technology, University of Malaya, Kuala Lumpur 50603, Malaysia; dmaliero@siswa.um.edu.my; 2 School of Computer Science and Electronic Engineering, University of Essex, Colchester CO4 3SQ, UK; 3 Tecnologico de Monterrey, Escuela de Ingenieria y Ciencias, Monterrey 64849, Mexico; m.zareei@ieee.org (M.Z.); cvargas@itesm.mx (C.V.-R.); 4 Department of Electronics, University of Peshawar, Peshawar 25000, Pakistan; arkhan@uop.edu.pk

**Keywords:** underwater acoustic sensor networks, UASNs, cooperation in UASNs, taxonomy of UASNs cooperation, game theory-based cooperation

## Abstract

Exploring and monitoring the underwater world using underwater sensors is drawing a lot of attention these days. In this field cooperation between acoustic sensor nodes has been a critical problem due to the challenging features such as acoustic channel failure (sound signal), long propagation delay of acoustic signal, limited bandwidth and loss of connectivity. There are several proposed methods to improve cooperation between the nodes by incorporating information/game theory in the node’s cooperation. However, there is a need to classify the existing works and demonstrate their performance in addressing the cooperation issue. In this paper, we have conducted a review to investigate various factors affecting cooperation in underwater acoustic sensor networks. We study various cooperation techniques used for underwater acoustic sensor networks from different perspectives, with a concentration on communication reliability, energy consumption, and security and present a taxonomy for underwater cooperation. Moreover, we further review how the game theory can be applied to make the nodes cooperate with each other. We further analyze different cooperative game methods, where their performance on different metrics is compared. Finally, open issues and future research direction in underwater acoustic sensor networks are highlighted.

## 1. Introduction

The Earth’s surface is 70% covered by water, where vast amounts of resources lie unexplored. Recent developments in sensor network technologies and emerging underwater sensor networks (UWSNs), have made the development of underwater applications feasible. The importance of underwater applications is attracting the attention of researchers which in turn has led to the rapid growth of research within various areas in Underwater Acoustic Sensor Networks (UASNs) [1]. UASNs comprise sensor nodes capable of sensing, processing and communicating the sensed data to the appropriate destination which are deployed underwater for an effort in backing vast range of applications in collaborative monitoring tasks [2,3]. Recently, many researchers are focusing on how to develop different underwater applications to support the lives of living organisms underwater and even those that are living on the sea floor by the use of collaborative, cooperative and frequent monitoring of underwater environments [4]. Recently, the frequent occurrence of disasters such as the sinking of USS ship name missing [5,6], the Kursk submarine disaster in Russia [5,6], the recent search for survivors of the Argentinian submarine ARA San Juan, and the Deepwater Horizon oil leak in the Gulf of Mexico [6], have encouraged several research teams to carry out various oceanic monitoring applications of UASNs such as oceanic surveillance applications [7], environmental monitoring, chemical exploration, military applications, and safety applications [4]. 

In UASNs, the exchange of information is the primary goal, and it can be achieved among the nodes within a stipulated network coverage area or outside the network coverage using a gateway device (e.g., sink). Underwater sensors are usually energy constrained since the nodes are battery powered. Therefore, to conserve more energy, transmitting packets in several small hops is helpful. By combining more underwater applications from private companies, government and non-government organizations, a single authority network would not be feasible. Therefore, the network can benefit from multiple authority network, where energy consumption can be reduced by cooperation between different authorities to forward the packets to the surface station. Also, packet delivery reliability can be improved and enhanced by cooperation between authorities. However, a new challenge may arise, where a single selfish authority or node can exploit the cooperation behaviors or in case of a malicious authority the data security can be threatened.

**Figure 1:**
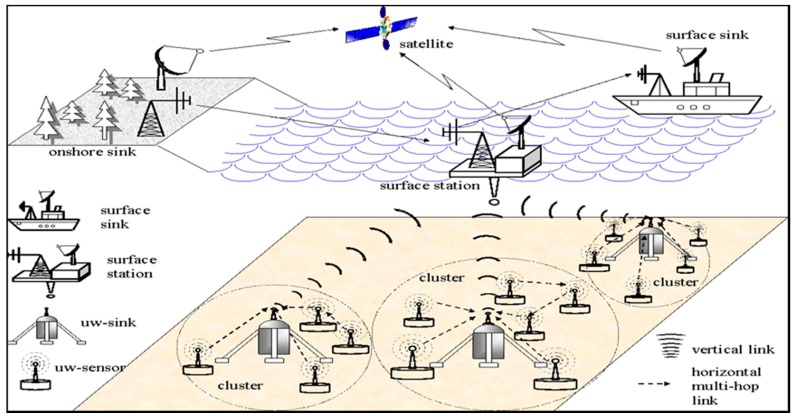
Cluster-based architecture of UASNs.

In these regards, cooperative communication techniques providing reliability in packet delivery for UASNs remains unexplored [8]. Acoustic waves are capable of being transmitted through the water to distances that are operationally significant. Because of this, sound is the physical phenomenon used for underwater communications. In underwater applications, an acoustic signal is a sound waveform which is usually produced by transducers. Even with noise and uncertainty, an acoustic signal processor is capable of extracting information from the acoustic signal [9]. In UASNs, sensor nodes are wirelessly interconnected with one or more underwater sinks (UW-sinks) via acoustic links. UW-sink can be connected with sensor nodes by two methods, either through direct links (DL) or through multi-hop (MH) paths [10]. These underwater sinks are responsible in relaying the sensed data collected from the sensor nodes situated in a particular location in the ocean to the surface station [11] as depicted in Figure 1 [11,12]. 

Each UW-sink is mainly equipped with some form of transceiver such as horizontal transceivers, which are used by underwater sinks for communicating the configuration data and commands with sensor nodes (UW-sink to sensor) and also collect the sensed data (sensor to UW-sink). Vertical transceivers are used by UW-sinks to relay the collected data to the surface station. Since the ocean can be as deep as 10 km, the range of vertical transceivers must be longer than that of the horizontal transceivers as used in deep water applications [11]. Parallel number of communications usually occur in multiple forms from the sinks that are deployed, therefore, to handle these issues the surface station has been equipped with an acoustic transceiver. The surface station can also communicate to the onshore sink (OS-sink) or surface sink (s-sink) using longer range of the radio frequency (RF) or satellite transceiver. There are many challenging factors which characterize the unique features of UASNs. These factors are:

(1) Sensor nodes are energy constrained because they are battery powered.
(2) The propagation delay of acoustic channels is five orders of magnitude slower than RF signals in terrestrial channel due to low speed of sound which is 1500 m/s.
(3) The bandwidth is limited and depends mainly on the distance because of high transmission loss with high frequencies and high environmental noise with low frequencies [13].
(4) Nodes are prone to failures due to corrosion and fouling.
(5) Fading and multipath problems make the acoustic channel impaired severely [2,3].

Formation of shadow zone and temporary loss of connectivity in a region where the reception of underwater signal is impaired due to fading and multipath. As the networks become highly populated and more congested, cooperative communication methods grow rapidly and gain attention as a way in which distributed nodes in sensor networks or ad hoc networks with multiple antenna systems can cooperate to achieve performance gains [14]. The unique characteristics of underwater channels make communication very challenging as the underwater nodes cannot directly use the protocols designed for wireless terrestrial scenarios [15,16]. In some underwater applications where autonomous underwater vehicles (AUV) exist, due to the high mobility of AUVs, cooperation within the nodes can be a major challenge which affects the overall performance of the network [17]. However, addressing AUV mobility challenge is not within the scope of this review.

Cooperative forwarding (routing) can be achieved when the nodes involved in the routing vector agree to participate and cooperate with each other. However, the possible existence of malicious nodes in the routing vector makes cooperation a very challenging issue. It is necessary to provide a suitable cooperative mechanism across multiple authority networks specifically designed for an UASN in order to achieve an effective cooperative performance among sensor nodes in UASNs. Underwater networks are more vulnerable to malicious activities due to their challenging characteristics such as high BER, high propagation delay, and low speed of sound, corrosion and bandwidth limitation with its dependency on both distance and frequency [18]. Although, several cooperative techniques have been proposed, more research is needed in the aspect of cooperation to guarantee the reliability of data packet transmission over a network between the sender to the target, as there might be possibilities of having malicious, compromised or selfish agents along the transmission path that would attack, compromise or damage data packets. 

In this paper, we aim to investigate game theoretic strategies to support node cooperation in UASNs, in order to secure and effectuate data packet transmission over multiple authority networks. A game theoretic cooperation evaluates players’ cooperation by modelling a two-person game and determine their level of cooperation based on their utilities (payoff) obtained in the game. Therefore, we have conducted an extensive investigation reviewing various factors affecting cooperation in UASNs where network consist of normal and malicious nodes. We study various cooperation techniques used for UASNs from different perspectives while concentrating on security and present a taxonomy for underwater cooperation. We further analyze different cooperative game methods, where their performance on different metrics is compared. Considering the game theory based cooperation, we extensively analyzed the most common methods and provide comparative tables of the existing methods. To summarize, the contributions of this paper can be highlighted as follows:

- We conduct a comprehensive review of different factors affecting cooperation in UASNs.
- Various techniques that are used in UASNs are presented and summarized from different perspectives and a taxonomy for underwater cooperation is presented.
- We further analyze different methods and compare their performance using different metrics in which our analysis shows that cooperative game theoretic based approaches are the most suitable and promising methods to provide cooperation in UASNs.
- We also discuss open issues and future research directions in UASNs.

The remaining parts of this paper are divided as follows: Section 2 presents the background on UASNs and related issues. A comprehensive review on cooperation in UASNs is presented in Section 3. Taxonomy of UASNs cooperation is described in Section 4. Section 5 presents the summary and discussion of the paper, and the conclusion of our work together with open research issues in UASNs and future research direction are presented in Section 6.

## 2. Underwater Acoustic Sensor Networks

The field of underwater acoustic networks has attracted a lot of attention in the research community, recently. Many applications such as environmental and pollution monitoring [2,19,20], oceanographic data collection [2], distributed surveillance [17], oceanic sampling [21], offshore exploration [2,20], disaster prevention [2], mine reconnaissance [2] among others are developed. However, there are many challenging issues that contribute to the UASNs performance degradation that include high energy consumption, loss of connectivity, security, routing and cooperation [20,22,23]. These issues in UASN are not well investigated in comparison to other applications such as those in Wireless Sensor Network (WSN) and Mobile ad hoc Network (MANET) applications. However, it is nearly impossible to apply those mechanism on UASNs due to their unique characteristics. Some of the distinguishing factors between terrestrial WSNs and UASNs are summarized in Table 1 and are as follows:

1. WSNs use Radio Frequency (RF) and UASNs use acoustic signals (sound waves).
2. Due to the low bandwidth and using high power amplifiers to produce significant acoustic pressure, UASNs consume more energy compared to its counterpart WSNs for terrestrial environment.
3. The high delay of acoustic signals is five orders of magnitude greater than that of RF in WSNs.
4. Bandwidth of UASNs is limited for some certain range and depends on both frequency and distance which is contrary to the WSNs.
5. UASNs suffer more from connection problems due to high BER and multipath problems compared to WSNs.

**Figure 2:**
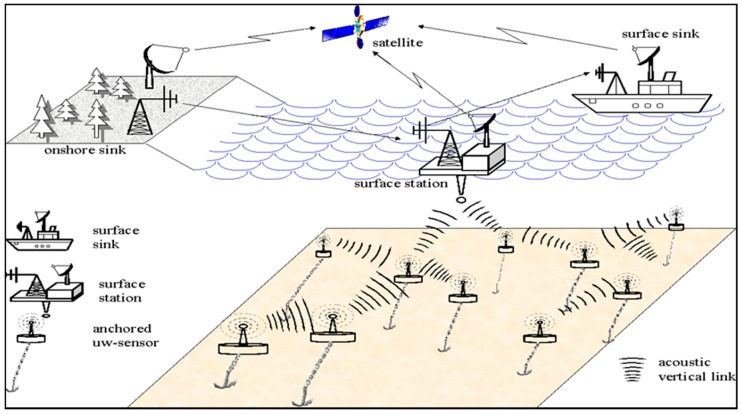
Cooperative-based architecture of UASNs.

Meanwhile, several works have been conducted targeting how to achieve cooperative communication, but reliability remains a major component of secured cooperative communication that is missing [24]. Cooperative communication can be achieved once the nodes involved in the source to destination path cooperate to support the delivery of packets. However, cooperative communication does not guarantee reliability of the data packets [25]. In fact, underwater data collection and reliable communication are challenging tasks. One of the promising solutions to these problems is to use underwater vehicles, which would move around the deployment area of the sensor nodes and collect their sensed data. However, planning the path for underwater vehicles to maximize the amount of data gathered and minimize the amount of time taken to travel in a particular region then becomes another challenge. The authors in [17] designed a behavioral approach based on cooperative forms of algorithms in which they used an underwater autonomous vehicle that is equipped with mobile sensor nodes. They were able to follow rules targeting a completion of a particular mission that is capable of maintaining the network connection link. Their proposed algorithm is robust and could improve the performance in terms of coverage and connection loss within the vehicle in underwater. It can also protect the communication from the denial of service (DoS) attack reactively. These types of cooperative algorithms are usually relied on availability of some information toward other types of sensors and such information may not necessarily be trustworthy and complete. However, sometimes they have been considered trustworthy for underwater oriented applications communication [17]. A schematic representation of cooperative based architecture for UASNs is illustrated in Figure 2 [11,12].

In [18], an automatic repeat scheme based on a particular request for communication in an underwater scenario is proposed, where they used cooperative device nodes for source–destination reliable path identification. It defines and provides alternative paths to certain connections of source–destination, which rely on the inter-node available information and the distance between source–destination and all other possible cooperative nodes available along the path. Upon receiving of any kind of packets that are erroneous (lost or dropped), the receiver would request the retransmission of that packet from the closest cooperative neighbor node . However, this has a high delay since at every time the node needs to buffer the packet for retransmission [26]. Thus, both the Automatic Retransmission reQuest (ARQ) and Stop and Wait (S & W) protocols suffer from long underwater propagation delays, therefore, they are not suitable for ensuring reliability in underwater cooperative environments that would result in better achieved performance. 

A cooperative technique which uses virtualization of sensor array from source–destination by adopting the benefits of Multi-input Multi-output (MIMO) systems in diversity space is proposed in [27]. This study adopts two protocols called decode and forward (DF) and amplify and forward (AF), which are cooperative protocols. When the relay node receives the signals, it decodes and forwards them to the destination node. As the source node sends the signal to the relay, it would decode them first and then forwards the decoded signal to the final destination in the DF protocol. Similarly, in the AF protocol, the relay node performs amplifying actions on the received signal and then forwards the amplified signal to the destination node. They provide two methods of transmission, first the direct transmission which is between source-destination and the second is signal transmission which includes the relay node [27]. 

A study of underwater AF and underwater DF that provides an improvement in the performance of BER in underwater for different configuration channels is presented in [25]. In this research, the focus is on providing reliable communication in which the authors study different underwater applications in three dimensions with cooperative transmission. Moreover, they introduce a hybrid method of forwarding in which the relay nodes have some flexibility and responsibilities of selecting the best scheme for transmission between underwater AF and DF. In order to forward the packet, a source node may look for and locate the relay node that would help the node by forwarding to a certain depth. Upon reception of the packet by the relay node, it would automatically adopt certain forwarding scheme based on the proper conditions of the underwater channels. Thus, these schemes do not provide a mechanism to select the appropriate relay node in a 3D underwater environment. Moreover, it does not take into consideration the energy consumption and the problem of having more than one relay node in the source–destination path. In [28] the authors introduced a cooperative authority method in order to maximize their energy underwater. It is demonstrated by different authorities that cooperation in forwarding the packet of other authorities achieves a maximum lifetime. Thus, the authorities that are selfish exploit the behavior of other cooperative authority. This method of cooperation was investigated by the concept of game theory, i.e., evolutionary game, in which they perform some simple analysis to show an achievement in cooperation even without an incentive mechanism. However, the major drawback of this work is not taking into consideration the mobility of nodes, which results in a dynamic topology and multipath. 

A study of network topology for multi-hop communication through diversity of cooperative transmission is introduced in [29], which demonstrates a better multi-hop performance as compared with its direct transmission counterpart. It also reveals that based on a certain distance and some predefined frequency range, multi-hop communication can achieve better performance in cooperative transmission, but this method suffers from multiple node interference (internode). In [30] a centralized position cooperative algorithm was designed which is used for automatic identification of a direct path, and it has demonstrated that it can efficiently achieve cooperative positioning for underwater scenarios. In [31], an autonomous distributed target tracking for cooperative sensor networks that works in two aspects is introduced. The first aspect is an intelligent system for gathering target information of autonomous vehicle behaviors using a control system, and the second aspect deals with a novel approach of multiple functions behavior-based objects using an autonomous vehicle control system with reactive control of multiple environmental constrains. However, it does not consider moving nodes (i.e., mobile sensor nodes) in detecting the target node and tracking vast number of sensor simultaneously, thus, it is not suitable for underwater applications. 

UASNs are vulnerable to malicious activities due to the communication medium. Several different types of attacks can be targeted on UASNs such as jamming attacks on the physical layer, acknowledgment spoofing in the link layer or sinkhole attacks and selective forwarding in the network layer.

**Figure 3:**
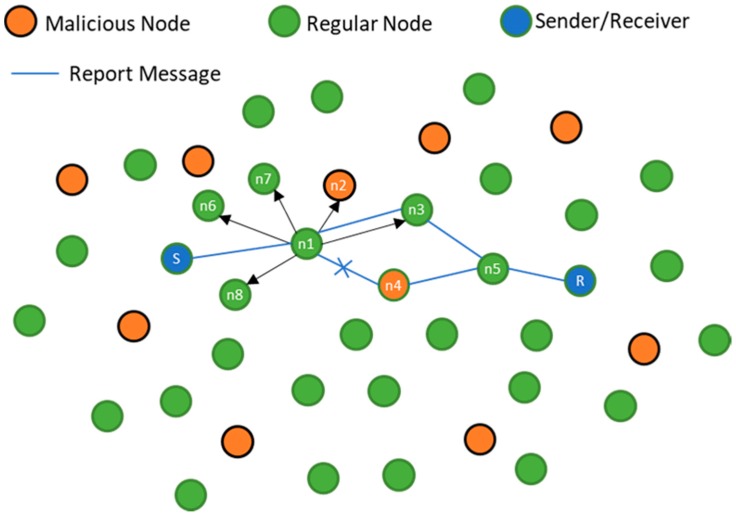
A Scenario with 32 nodes with 10 malicious nodes.

Figure 3 illustrates how malicious nodes affect the performance of cooperation in UASNs [22,32]. In this scenario node S wants to send message to node R. Node S chooses to send the packet to node n1 as a relay which is inside the routing vector toward the destination. Node n1 initially forwards the packet to n4, and it happens that n4 is a malicious node and n4 drops the packet. Node n1 notices that n4 is malicious, thus n1 sends a message to all its neighbors that n4 is malicious, and n1 selects n3 as a forwarding node which relays the packet to R (destination) [32].

Game Theory-Based Analysis (GTBA) proposed by [33] is an incentive-based cooperative method designed to solve the cooperation issues among regular nodes and malicious nodes. However, GTBA has not considered several attacks such as malicious node attack which makes unsuitable for UASNs. In [24], authors proposed a routing protocol for secure routing in which they utilize the primitive cryptographic concepts for the UASNs mobile nodes and fixed nodes. In [32] authors proposed a Dynamic Bayesian Signaling Game (SRPDBG) model to enhance the routing security to achieve a successful packet forwarding among nodes where they analyze regular nodes and malicious nodes. However, their approaches did not address cooperative mechanisms for end-to-end packet delivery in UASNs.

A game theory approach described in [34] provides a solution to the problem of network security in MANETs which prevents conflict between sensor nodes with high mobility in terms of cooperation. The similar approach is applied in various fields such as computer networks, political science, behavioral biology and economics. In another work, a vector machine in support of attack detection and identification of malicious nodes in underwater wireless sensor networks (UWSNs) was proposed by [18].

Souza et al. [35] discussed an energy evaluation scheme for varieties of digital signature approach to achieve authenticated forwarding and discussed suitability of using digital signature for UASNs despite its challenging features and characteristics. They adopted the concept of cryptographic mechanism of PKCs to achieve confidentiality and authentication. They also evaluate varieties of schemes for digital signature used in UASNs, where they classified them as short and long signature, however, the scheme only focuses on the efficiency of power consumption [35].

## 3. Cooperation in UASNs

**Figure 4:**
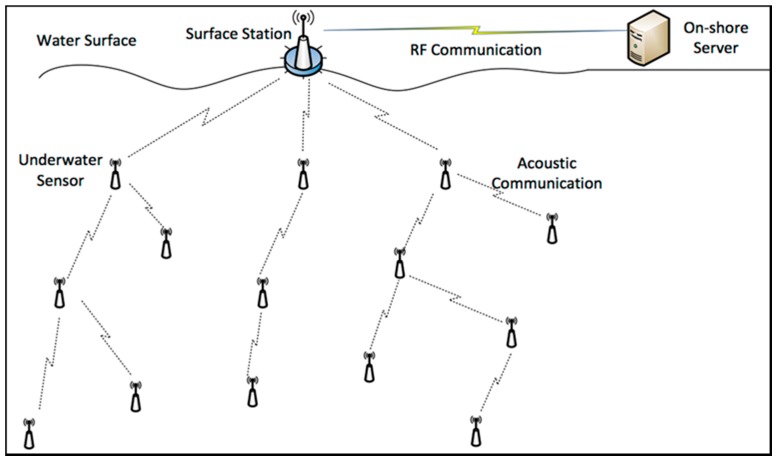
Cooperative multi-hop UASNs.

Cooperative communications, in UASNs, remain a challenging concept due to the unique features of UASNs, thus, some of the protocols designed for MANET and WSN cannot be directly applied to the underwater environment. Researchers in [14] conducted an analysis for propagation error in underwater cooperative multi-hop communication, where they examined the expected gains of multi-hop communication. Cooperative multi-hop communication in UASN is illustrated in Figure 4 [36].

A data collection method based on coded cooperative OFDM for an underwater dynamic network is developed by [37], where a mechanism of coded cooperation based on selective relay and dynamic network protocols were proposed. The study of least square cooperative localization for UASNs was proposed by [38]. In their approach, the performance metrics like consistency, coverage and efficiency were analyzed in cooperative localization least square to quantify the localization accuracy deviations.

However, the authors proposed distributed algorithms for least square cooperative localization in which the squared-range is integrated by passing the message to a localization network. Moreover, Ref. [39] proposed paths for data collection of mobile node for cooperative UASNs in order to increase the efficiency of data collection for nodes on the surface station. In [17], an autonomous cooperation of mobile sensors in UASN has been studied, where a novel cooperative algorithm is presented in which every autonomous underwater vehicle (AUVs) is equipped with mobile sensors. Moreover, the algorithms take into consideration the maximum communication constrain between the agents based on communication range. A motion algorithm based on the control is decentralized to enable every vehicle to perform some maneuvers in order to achieve minimal error of acoustic source localization. Every autonomous vehicle in an underwater environment is equipped with a hydrophone array custom designed for it to perform some bearing angle measurements between an acoustic source and an array. 

Acoustic cooperative transmission for underwater communication that utilizes cooperative communication is proposed by [40], where various schemes of transmission for cooperative underwater communication which are specifically designed for communication that uses radio signal were analyzed. By considering the sound speed they also developed a new cooperative scheme for wave transmission, where they applied the concept of AF protocol along source–destination. These cooperative underwater transmission techniques reduce the limitations of the channel and improve the acoustic underwater channel throughput. 

The acoustic channel is characterized by long propagation delays and high BER. Therefore, it is challenging to design an efficient energy consumption method to achieve reliability in data transfer for time based applications. To overcome this issue authors in [41] proposed the Multi-path Power-control Transmission (MPT) scheme that improves the performance of the network in terms of energy efficiency, delay and low error rate of packet forwarding. MPT uses a scheme that combines the method of power-control with the method of multi-path forwarding. Moreover, the destination combines the packet received and the storage is designed for power-control. As it does not require retransmissions of packets, it uses less energy compared with its counterpart schemes using single path transmission. MPT also achieves less delay since it does not use a hop-by-hop transmission scheme. However, MPT does not solve the long delay problem of UASNs. 

A cluster method for achieving cooperative communication is studied and analyzed by [42] where they use sensor nodes in every cluster for gathering of data related to a particular deployment area and forward the gathered data to the cluster head (sink) in a collaborative and cooperative manner. Several sensors are modeled as a dependent variable that are randomly dependent on both realization of noise and channel in which the optimization issues of multi-variable is formulated for minimizing the total consumption of energy. Moreover, an investigation for energy efficiency which is affected by power transmission allocation was conducted. However, the method can only work on a homogeneous network with the same parameters. Acoustic distributed monitoring was implemented in the novel work of [43] that is based on storage of sensors and a trace system of data retrieval for a particular application domain which is designed for recording or storing of data in acoustic networks. They used a scheme of recording in a cooperative pattern and a mechanism of storage balance distribution for resolving the challenges of the large volume of storage and acoustic sampling with high frequency, but this scheme does not solve the unreliability problem for underwater communication. 

A system of cooperative storage developed by [44] aims to maximize the overall storage of a distributed node system that is disconnected. It uses data mules to forward the data among nodes in an opportunistic network. However, the method does not consider the collaboration of several mules for cooperative underwater monitoring. Moreover, the policies for the replacement of data to have flexibility in the information, not only the data which is already stored, is not considered in the paper. Analysis of multi-hop cooperative propagation is presented in [14] in which they study the most common issues of propagation errors in multi-hop and compared it with its Direct Transmission (DT) counterpart which reviled that Multi-Hop (MH) can provide higher cooperative underwater packet forwarding than DT. This analysis was conducted using a Markov grid and linear networks, however, as the scheme does not work with a multi-channel environment it is inadequate for underwater scenarios. In [45] the authors proposed sensor robot cooperative techniques for underwater monitoring activities. They designed an architectural based system to provide control support for power navigation using cooperative algorithms, mulling data application and cooperative experimental navigation characterization that support long monitoring of underwater robots. A survey of comprehensive localization schemes is presented in [46] that classified the algorithms based on three components of the mobility in the sensor called mobile, stationary and hybrid algorithms. Furthermore, an analysis and comparison of algorithms for localization was performed to determine the direction of future research in underwater. However, they did not cover collaboration among nodes that may result in cooperation that makes it unsuitable to achieve cooperation in UASNs. 

Location identification of sensor nodes in UASNs is an important aspect of cooperation. In this context the authors in [47] introduced a free anchor localization method for UASNs. They analyzed and concluded that the existing methods using GPS are not suitable for underwater localization. They also designed UASNs active-restrict to solve the problems of GPS which does not require the node information, but uses the node adjacency relationship for localization and is used in dynamic and static networks. However, there is no cooperation between distinct characteristics of communication in underwater and it is also not suitable for reliable cooperative communication in an environment like underwater. In this section, we provided a broad view of cooperation in UASNs. We have discussed some related works and the weaknesses of each method. In the next section, we take a closer look at the cooperation in UASNs by categorizing the existing works and analyzing them in more details.

## 4. Taxonomy of Cooperation in UASNs

**Figure 5:**
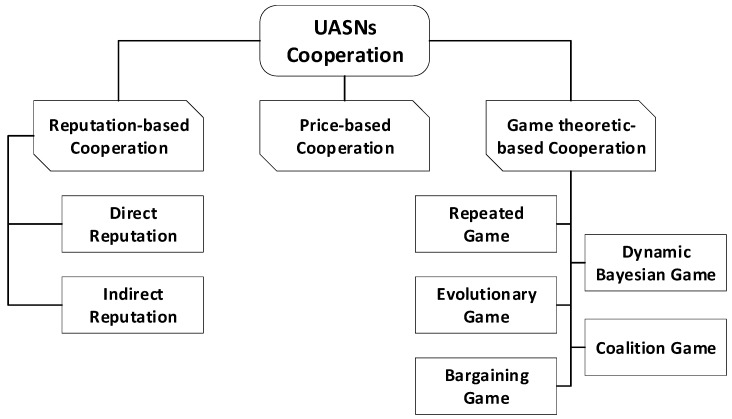
Taxonomy of cooperation in UASNs.

The taxonomy of cooperation in UASNs is presented in Figure 5, where it is classified into three main categories which are reputation-based, price-based and game theory-based cooperation. This review mainly focuses on the game theoretic-based cooperation aspect, providing a thorough investigation on the current literature in cooperative game theory applied to UASNs to reveal the most suitable cooperation techniques to ensure reliable cooperation in UASNs. 

### 4.1. Reputation-Based Cooperation

Reputation is a mechanism used to determine the trustworthiness of an individual node that is involved in an ad hoc routing process. It is employed in various fields of underwater research that require successful packet forwarding among nodes. It detects the historical behavior of the opponent for decision making as well as future behavior expectation prediction under uncertainty based on trust [48]. It has been used for different scenarios to demonstrate the degree or level of trust among the variety of participants in a cooperative domain, and to apply some punishment methods for non-trustworthy nodes based on some stipulated threshold level. It can also be applied in the determination of the trustworthy routes, which do not contain malicious nodes in the route [49]. A reputation system cooperation can be achieved by evaluating the trustworthiness of each node using the behavior of such node and use the reputation values to detect the misbehaving nodes. Every node can maintain their reputation table record regarding the other nodes reputation values, where they used a threshold to differentiate between the cooperative nodes and misbehaving nodes [50]. Cooperation can be achieved when the involved node attains the high reputation values (cooperative node) and whenever the node obtains a value lower than the threshold, it is considered as misbehaving node. Every cooperative node with high value of reputation can be selected as a relay for routing a particular packet to the target [51].

#### 4.1.1. Direct Reputation (First-Hand)

A direct reputation (first-hand) is a system in which a particular node observes the behavior of other nodes, and based on the observed reputation, the node calculates the reputation value of other nodes and store it in the reputation table. The node punishes the nodes with low reputation value (below the threshold) that are detected by isolating them from the network and not using them for information exchange based on a particular reputation system [52]. In [53,54], authors allow a particular node (ender) to select the relay node that has high reputation as the next-hop in order to route the packet to the target node to achieve maximum cooperation and reliability. In OCEN [53] they do not accept (second-hand) indirect reputation observation forms of information, but rather they use a direct observation to achieve a secure cooperation. In [55] authors introduced the idea of expanding the node behavior observation scope to more than one hop.

#### 4.1.2. Indirect Reputation (Second-Hand)

An indirect reputation is introduced by [56,57] that allows the periodic exchange of the observed behavior of a node with other nodes. In CONFIDENT [58,59] and CORE [56] there is a monitor module that continuously observes the behavior of the neighboring nodes in the network. This module detects the misbehaving nodes and broadcasts an alert about them to other nodes. When other nodes receive such alert, they delete them from their list; so that, they will be gradually isolated from the network. Although, most of the common methods of adjusting the reputation can be either by linear method [55] or by nonlinear method [57], the selfish clever node may maintain a high reputation threshold by forwarding other node’s packet which makes it undetected. In [57], a reputation-based method that uses direct reputation information so that each node can maintain the rating and information about the reputation of other nodes is introduced. Furthermore, they designed a reputation-based approach which takes into account the indirect reputation and accepts the modified reputation rating information. A reputation information-based rating with indirect reputation compatibility for trust updating and rating is proposed in [60], where the historical behavior of the opponent node is captured and the expected future behavior for such opponent node is predicted by the use of trust. In reputation based approaches, usually two forms of information are obtained, one is obtained by interacting directly to the neighbor (intermediate) and the other is obtained by the direct neighbors’ aggregation.

### 4.2. Price-Based Cooperation

A price-based cooperation is one of the classifications of underwater cooperation that relies on the price (i.e., virtual currency) system in forwarding the packet form source to destination via intermediate nodes. Price-based cooperation techniques consider forwarding packets similar to transaction services which use virtual credits (virtual currency), in a way that every node that provides the forwarding services should be “paid” by the node that receives the service [60]. In price-based methods, a node that requests the service should pay the node that provides such a service and the payment can be in the form of points, money or any kind of valuable objects [61,62,63,64]. Nuglet is one of the price-based methods introduced in [61], where every forwarding action requires credit in the forms of nuglets for managing transaction services. A study to determine how such credit should be used and evaluated is investigated in [63] which identifies the method to evaluate the price to encourage cooperation among nodes in UASNs. In [65], authors proposed a defense method to avoid cheating in providing forwarding services, i.e., a node may receive the credit and deny service or request a credit for fake services. In [62], an analysis of incentive scheme based on the trust management for allocation of resources problem for collaborative network in a context of intrusion detection is introduced. They formulate the related and relevant cooperative game where they obtain the Nash equilibrium of the game by using their proposed dynamic algorithms.

### 4.3. Game Theoretic-Based Cooperation

Game theory is a well-developed field of mathematics which is a suitable way to analyze outcomes of group behavior players are basically rational. A rational player chooses an action that maximizes its outcome given its belief about other players’ preferences. The game analysis predicts the final outcome when rational players play against rational players. They provide information and instructions to determine the attacks, but they are unable to provide concrete solutions to the identified problems [66,67,68,69,70,71]. In [49], authors study various game theory approaches and their impact on network security applications. They provide solutions to a vast number of problems in network security based on an approach based on the theoretic concept of games. The authors in [69] classified and categorized various number of attack strategies against UASNs. According to [48] “reputation- based Bayesian game is type of game described as a non-zero-sum game where players can compute payoffs for each action based on reputation and estimated degree of the opponents”. 

#### 4.3.1. Repeated Game or Decision Making-Based Games

Game theory uses mathematics to express the phenomena of decision making among more than one agent [68]. It focuses on providing a mechanism for optimality in the network as well as providing reliability in the cooperative routing and forwarding the packets. All players in this type of game are usually assumed to be completely biased in that they only focus on their expected gain in the particular strategic game [72]. The authors in [73] use game theory to model the interactions between a society of agents as a repeated game. They analyzed distributed search for resources in networks where agents interact with each other and offer services and consume these services. They have associated a cost to every agent in a non-homogeneous manner to study which network structures are more appropriate to promote cooperation. These forms of security problems are mostly addressed in the forms of attacker (i.e., malicious node) and defender which can be either transceiver or the receiver using a repeated game. It is a decision-making (DM) game such that a DM has advantage or information about the opponent player. It can also be a collaboration toward achieving a particular target if the decision making cooperates in a coalitional or cooperative game. The game of interaction among players can be modeled in a way to improve the privacy of both players in the game. A game of incomplete information can be modeled to evaluate the issues of the repeated game, which can contain simultaneous movement of players. Because these incomplete information games are dynamic, there is always a trade-off in terms of privacy and trust in the game of theoretic concept which is based on the trust formulation [34]. Some forms of game classes can be applied to the dynamic uncertainty in which the domain of uncertainty can be modeled and represented as a decision process in the game [74]. An algorithm for path planning based on scheduling proposed in [75] overcomes the Traveling Salesperson Problem (TSP). It provides a multi-node communication and improves the performance of path selection. To achieve this, there is a need for a robust protocol for scheduling that solves the problem of channel interference and variation. Mathematical comparison of repeated games approaches is presented in Appendix A. Repeated games permit a drastic defect that allows the players to behave selfishly and obtain maximum payoff (Nash Equilibrium), and there is no sufficient punishment to the selfish participant, which makes it not suitable to encourage cooperation in UASNs.

#### 4.3.2. Dynamic Bayesian Game

In [32,76] the authors proposed an enhanced secured routing scheme using a concept of signaling game in dynamic Bayesian model in which they analyze the regular strategy profile of malicious nodes and how to protect the nodes from anonymous behavior. The authors in [77] proposed a Secure and Distributed Reprogramming Protocol (SDRP) which uses identity-based cryptography to reduce the storage requirement and communication of every node based on secure reprogramming. A convergence in terms of true cooperation for Bayesian game was designed in [48], which uses the concepts of reputation values to analyze the payoff for the game. They define two different types of participants, honest type and dishonest type. They illustrate the sustainability of true cooperation based on repeated game even on the dynamic application, and there is an increase in the average players’ reputation over a given time in a certain coverage.

A routing model based on the concepts of a dynamic Bayesian game was proposed by [78] where they analyzed routing concepts theoretically to fill the gap between decision making of non-simultaneous and information history which is also incorporated in the process of theoretically routing modeling in the approach of game theory. However, the problem with this approach is that it does not provide the practical test of different functions of utilities and the probability distribution in the game on different parameters of networks such as node mobility rate and selfish node in order to have fair comparison with existing works. A scheme for secure and robust routing is proposed in [79], where an interaction between sender node and receiver node was modeled on the basis of a game model. In this work, a dynamic Bayesian game were used based on node opinion about the opponent in which the destination node established the mechanism of acknowledgement for the reception of the packet from a participating node in the game. Jiang et al. [80] proposed a scheme based on the game theory of interaction, coding theory and establishment of trust for the restriction of attacks and colluding. In these schemes, the message availability is guaranteed whenever there exists a legitimate path. This scheme also shows that it achieves efficiency in terms of latency, energy consumption as well as availability of paths, but the scheme does not provide reliability of message delivery among nodes.

In [81], Jin et al. studied, analyzed and addressed a vast number of privacy and security problems for different applications in computer science such as mobile and network applications, where they organized their work into several modules that addressed a particular problem in the aspect of security. They also designed a mechanism for security and conducted an analysis under equilibrium, where they highlight the advantages and disadvantages of game theory. However, the work only covers the theoretical part, which is helpful in terms of developing solutions to the problem of security of the network using a game theory approach [34]. An access based on point pricing modeling for dynamic game is proposed in [82], where they modeled a two-person game between the access point owner and client, in which both have some set of symmetric information. However, the client has more information than the owner of the access provider. It is found that a client has a utility function which is the web browser and the Nash equilibrium which enables the provider to take advantage of the client and change the constant price charge for the client for a given unit of time. A majority of the security game classes define a two-person strategic game where one of the players would act as an attacker trying to hack and damage the system in order to degrade the performance. However, in this study the game is different due to the fact that the game is an n-person strategic game, where all the players are rational, and these players always prefer to take actions that would benefit them. Mathematical comparison of Bayesian games are presented in Appendix B and Appendix C, where the papers in Appendix B take all the cooperation metrics into consideration such as payoff calculation, player strategy profiles, player best response, player belief, payoff matrix and Nash equilibrium, and the papers in Appendix C only considered three cooperation metrics such as payoff formulation, player belief and Nash equilibrium. This class of game is the most suitable for maximizing cooperation in UASNs since players must cooperate with their opponents to obtain maximum payoff (utility). The degree of cooperation among the players is always equal to the degree of their payoff. Therefore, the more players cooperate the more degree of payoff would be and vice versa. All the players of this game are assumed to be rational and they only focus on maximizing their expected payoff at all times.

#### 4.3.3. Evolutionary Game

In [71] an Evolutionary Game-Theoretic (EGT) approach is proposed, where they study trust decision and its dynamics to illustrate the evolutionary process of sensor nodes (SNs) in selecting their action and approaches that can lead the SNs to choose the action trust as their final behavior. The authors in [51,83,84] proposed an intension recognition-based method that employs a repeated dilemma special context and evolutionary game, where the internal dynamic trust of intention recognizer and opponents were assessed. Moreover, they use direct interaction method of the past to predict their opponents’ next move, and as a result they are able to prevail on the famous cooperative strategy of the repeated dilemmas. A model of adaptive trust on the basis of Request as well as Authentications (ATRAM) was proposed in [50,85], where a game theory and the information history of interaction within peers is employed. The proposed scheme guarantees secure data transmissions in a mobile peer-to-peer network regardless of whether each of the peer node can have access to the trust information about the peer and risk information about the other peer or not. The scheme has been further categorized into four types of models that are called game-based, trust-based, risk-based and role-based. In [86,87], an analysis of game theory which defines the factors of adaptive and cooperation development using EGT is proposed. It utilizes the assumption for zero-state information and non-zero state information between peers (i.e., trust value and risk). The designed model ensures that the priority is given to requested peers for a connection with a peer of resource. However, due to trade-off in terms of performance and security aspects, this model does not adequately address reliability of data packet among nodes. A comparative table of equations applied in evolutionary games is presented in Appendix D. Moreover, in this class of games rational behavior of a player can be possibly combined with pure strategy for enhancing the characteristics of their population (authority or group) and since players are not rational players this method is not suitable for UANSs cooperation.

#### 4.3.4. Bargaining Game

In a bargaining game, the problem is usually modeled and represented as two agents (competitors) that might cooperate with one another. This game is also known as a Nash Bargaining Game (NBG) which is modeled on the concept of an interaction bargaining among two agents (players) that would seek (request) for the benefit of the same fraction [88,89]. Resources allocation in a communication networks can be modeled using bargaining game, where the players are targets to exploit a spectrum for the same benefit that would be allocated among the players fairly. In NBG, whenever there exists a number of requests that exceeded the resources that are available from the two agents or players, such requests would be discarded. However, if there exists a request from those agents that does not exceed the available resources, such requests would be completely accomplished. Pareto-inefficiency occurs because of non-cooperative players which is solved in NBG solution. In the method proposed in [90] the resources must be enough to satisfy both of the players’ requests. When all their requests are granted other requests would be discarded and this is the main problem with bargaining game. Hence this class of game does not provide suitable cooperation in UASNs. A comparison of bargaining games is presented in Appendix E.

#### 4.3.5. Coalition Game

A player’s best response is an action chosen by the opponent player which would lead or enable the player to maximize his/her payoff or achieve maximum payoff or Nash equilibrium of the game (coalition value) [88,91]. A Nash equilibrium is a vector in payoff matrix where both the players have achieved a maximum payoff (i.e., all players reach their highest payoff). A player’s best response can only emerge if and only if the action chosen by an individual player results in the maximum payoff or expected gain of the opponent player [92].

Due to the facts that both players are assumed to be rational, they are expected to be biased in selecting actions that will favor them to have a maximum outcome at the end of every round in the game. The players are biased in the sense that they always consider themselves as a priority [24,93]. In coalition forms of game, it can either be in partition forms or strategic form. In a partition form of coalition, the players participant number would be determined by the coalition value which is regardless of establishment of the network [72]. A comparative table of equations applied in coalition games is presented in Appendix F. However, this class of game depends on how many participants are playing the game (coalition depends on participants) instead of the establishment of the entire network, which makes it not suitable for UASNs cooperative communication (routing).

## 5. Discussion and Summary 

In this work, we have considered the cooperation issues in UASNs that would degrade the performance of the network which is mainly attributed by the presence of malicious or misbehaving nodes in the routing vector (source-destination path). We conducted a comprehensive review for investigating different factors affecting cooperation in UASNs. We study various techniques used in UASNs from different perspectives and present a taxonomy for underwater cooperation. We further analyzed different game methods and compared their performance using different metrics, in which our analysis shows that cooperative game theoretic-based approaches are the most suitable and promising method for providing cooperation in UASNs. A comprehensive summary of different game approaches is presented in Table 2, which summarizes the addressed problems, the applied methods, the main contributions and their limitations. In summary, based on our study there is no work that has high performance in all the metrics we have analyzed as shown in Table 3. In Table 3, a Yes means the paper considers that issue and solves it to a certain level (degree), and No means that the paper does not, in any way, solve that issue.

An analysis and evaluation of existing literature works is highlighted in Table 3 where many metrics are compared and it is demonstrated that there is a gap which need to be filled in order to achieve maximum effective, efficient and reliable cooperation among nodes in UASNs. However, there is a need for providing an efficient cooperative mechanism that would take all the performance metrics into consideration by maximizing packet delivery ratio, coverage, selfish detection, security and reliability, at the meantime minimizing energy consumption, delay and routing overhead.

## 6. Conclusions, Open Issues and Research Directions

In this work, we have conducted a review investigating different factors that affect cooperation in UASNs. We study various techniques applied in UASNs from different perspectives and we present a taxonomy for underwater cooperation. We further analyzed different game methods and compared their performance based on different metrics in which our analysis shows that a cooperative game theoretic based approach is the most suitable and promising method for providing cooperation in UASNs. Games will let the network nodes decide the best options based on interaction through game rules, so a malicious sensor would need to interact using the game rules, and that interaction would not necessarily result in a successful transaction. In contrast, reputation- and price- based techniques would not be so effective in scenarios where malicious nodes come and steal identities from good nodes, since they only need to announce their reputation or price and interchange messages to be considered as reputable nodes. The competition included inherently in games would make it difficult for a malicious node to get trust from other sensors.

Competition brings the best result for all the players, that is why the use of game theory provides a platform for competition under a framework of rules, whereas the other two techniques (reputation-based and price-based are based on bonuses or punishments, thus creating an environment where individuals try to excel (having the highest reputation, or having the best price of a service to be used by others), not necessarily in a cooperative way that benefits more individuals. Game theory on the other hand creates these environments where cooperation achieves a common goal or network objective without the interest to excel individually.

Through our literature review, we have found that none of the current research considers all the performance metrics that are necessary for an optimum network performance. There are many aspects or components which support and contribute to better achieving cooperation performance in UASNs, see last column of Table 2. Therefore, the exploration of those components is yet to be addressed in future research as an integral part of the cooperative game. A suggestion of some of the necessary aspects of the network that need to be included in cooperative game methods and need to be studied further are:

- *Reliability*: This is one important aspect to have a reliable successful forwarding and delivery of information among participating players of sensor node in UASNs. It is a key point that guarantees various forms of reliability such as data reliability, hop-by-hop reliability and end-to-end reliability. Cooperative game methods need to consider strategies such as information protection, packet repetition, route redundancy and maintenance, to provide reliability. This means that cooperative methods to be developed need to be extended to include reliability issues such as those mentioned at the same time of performing cooperation. Reliability guarantees a successful delivery of data between players participating in cooperative or collaborative activities. The investigation in this research found that this very important key component of cooperation is missing in most of the current literature in UASNs, and therefore, it is required to come up with a cooperative mechanism that will take this reliability into consideration.
- *Efficiency*: Efficiency is strongly required in a communication network to provide an efficient cooperative method and to facilitate cooperation among players. Based on our investigation of the current work, it has been found that there is no scheme, method, approach, or mechanism which takes into account this aspect. Cooperative or collaborative monitoring activities require an efficient mechanism for a successful packet forwarding and delivery in UASNs. Efficiency needs also to be included in cooperative game techniques to use resources that ensure an efficient delivery of information, if not, then the cost of such information delivery will increase, i.e., delays, throughput, integrity of information, etc. The cooperative game methods used that integrate reliability and efficiency, can establish a base on which a quality of service (QoS) model for UASN can be built.
- *Motivation*: Since misbehaving (malicious/selfish) that would try to damage, attack, and compromise or degrade the performance of the system nodes might be present in the network, a motivation mechanism is needed to influence and change the behavior of such nodes. Motivation is a way to make nodes react to conditions in order to get more cooperation or to improve network performance. Games have always motivated people based on competition, hence, such motivation can be included so that sensors participate in more cooperative actions for the improvement of network performance. Motivation is very important in cooperative forwarding activities, and providing motivation to misbehaving nodes in UASNs becomes necessary to ensure a successful delivery of data packets among the participating players. These motivations will improve the cooperative performance of regular and misbehaving nodes which will result in improvements in the cooperative packet forwarding processes in UASNs.
- *End-to-End authentication*: End-to-End authentication can provide security to the packet forwarding process among participating nodes, and can prevent the data in the packets from being compromised by devious misbehaving nodes. Authentication is important in any network, but when cooperative games are involved, it can be achieved in several contexts. For example, it can be used to authenticate locally in a network by using only previously authenticated one-hop network nodes, but the cooperative games allow to also implement authentication from the perspective of an end-to-end connection. Cooperative games need to be modified to address this context because the process of end-to-end authentication could be accompanied at the same time by reliability (e.g., routing redundancy or delay), by efficiency (e.g., use the right resources that ensures quality) and by motivation (e.g., route competition to choose the best path), in order to deliver a solution with quality to the transmission of information in the network. Authenticating data before forwarding it to the target destination is an important aspect of cooperation that guarantees the security of information forwarding between source and destination in a communication network.

## Figures and Tables

**Table 1:** Comparison of WSNs and UASNs.

Comparison Parameters	WSNs	UASNs
Communication Method	Radio Frequency (RF)	Acoustic Signal (Sound waves)
Energy Consumption	Low	High (due to energy cost of submitting packets)
Propagation Delay	Low	High (five orders of magnitude greater than WSNs)
Bandwidth	High	Low (depend on distance & Frequency)
Connectivity Lost	Low	High (due to high bit error rate)

**Table 2:** Summary of cooperative game methods in UASNs.

Paper	Problem	Method	Contribution	Weakness
[50]	Problem of one hop cooperative packet forwarding due to noise a packet lost	Evolutionary game theory with dynamic strategy of cooperative	Guarantee the cooperation convergence by driving the ratio of cost-to-benefit threshold	Do not focus on any security issues and hence no mechanism for malicious node detection
[51]	Dynamic trust cooperative under high malicious ratio	Evolutionary game-based trust cooperative simulation model	Convergence optimal strategy which maximize payoff and trust strategy preferential	Reliability issues in the packet delivery has not been tackled
[59]	Analysis of cooperative incentives	Integrated system of Game theory	Detection of selfish node and Effective cooperative incentives	The security issues, attack or node that are compromised has not been addressed
[33]	Regular and Malicious Node Strategy Analysis	Dynamic Bayesian Signaling Game	Formulation of Bayesian game framework to study the strategy of regular and malicious node	The game does not solve multi attack collision of regular or malicious node
[24]	Security issues such as confidentiality and integrity in underwater	Cryptography method and Secure routing	Practical and efficient to confidentiality and integrity bin UASNs	It does not fully encourage cooperation in UASNs
[32]	Lack of centralized control and secure routing	Dynamic Bayesian Signaling Game	Formulate a two-player game and analyzed the Nash Equilibrium strategy	These is no any suitable mechanism for long running game to solve various attack issues
[72]	Preventing malicious attack and Assuring trustworthiness	Evolutionary game theoretic approach	Prevent nodes form attack and guarantee trustworthiness of data	These is no much impasses on cooperation issues
[70]	Consumption of resources and selfish detection	Bayesian Game Method	Performance improvement in recourse consumption and effective security	These is no motivation to cooperation enforcement
[71]	Trust evaluation and Trust decision issues in individual strategy adjustment	Trust Strategy based Evolutionary game model	Data retransmission after Packet lost, build a trust strategy and strategy adjustment	No trust value calculation and trust management for security basis
[40]	Interaction for decision making and malicious behavior of entity	Bayesian Game model with dynamic repeated type	Motivation to answer the request trustfully and promoting node to be honest & cooperative	No adequate solution to the selfish detection and therefore is not secured
[81]	Unknown malicious Selfish, nodes cooperative & trustworthiness	Bayesian Game theory model of TPP	Trustworthiness of unknown node evaluation and drive the equilibrium strategy of the game	TPP game is not a multi-player game and hence cannot handle multiple payoff game
[28]	Cooperation between different authorities to reduce energy consumption and maximize lifetime	Evolutionary game theory with reactive & non-reactive strategy	Show Cooperation can emerge underwater without incentives and highlight factors affecting cooperation and the way they affect it	Did not consider propagation in multipath & dynamic nature of underwater topology
[35]	Authentication problem for security issues	Digital signature scheme	Energy cost evaluation using digital signature scheme (end-to-end) authentication	Lack of cooperation mechanism among the participating node
[83]	Behavioral evolution interaction recognition	Bayesian network model by Repeated Prisoner’s Dilemma (PD) and evolutionary game theory	Assessing the internal dynamic trust between intention recognizers and their opponents and predict the next move of their opponents based on the past direct interaction	Intention recognition achieved high performance of cooperation in homogeneous network only
[21]	Illegal accessed of transmission in underwater communication	Iterative key distribution scheme with secure routing method based on the Focused Beam Routing Protocol	Reduce the redundant keys in the key distribution system and adopt the mobility model to capture the movement of sensors node floating on the sea	This scheme did not consider the impaired channel caused by path loss, noise, multi-path and fading in underwater environment
[48]	Centralized and distributed reputation management in massive number of entities.	Bayesian Reputation Game	They found that trust cooperation is sustained when the game is repeated and the average reputation values of the players increase over time and coverage	The approach does not focus on the security issues among nodes and hence is not reliable
[59]	Investigating the effectiveness of nodes cooperation incentives	Used game theory to analyse cooperative incentive by Integrated System which combine the Reputation system and Price-based system	They found that the strategies of using threshold to find the trustworthiness node in Reputation system and Price-based system can be manipulated by clever or selfish node. Integrated System achieved high performance in effectiveness of incentive	The limitation of these method is it does not provide solution to the security issues among nodes
[60]	Motivate to share services and resources and to avoid selfish nodes to hinder the functioning of the entire network	Virtual currency and reputation mechanism method	They exploit the willingness of member to share their resources/services in order to increase collective welfare and to extend the reach of existing infrastructures	Their scheme does not guarantee security of data, since a selfish or clever node can manipulate the threshold value

**Table 3:** Evaluation and comparison of the approaches based on the performance metrics satisfied.

Paper	Energy	Delay	Routing Overhead	Packet Drop	Selfish Detection	Coverage	Security	Reliability
**[59]**	No	No	No	No	Yes	Yes	No	No
**[24]**	Yes	No	Yes	No	No	Yes	No	Yes
**[32]**	No	No	Yes	Yes	Yes	Yes	Yes	No
**[72]**	No	No	No	Yes	Yes	No	Yes	No
**[21]**	Yes	No	Yes	No	No	No	Yes	No
**[70]**	Yes	No	Yes	Yes	Yes	No	Yes	No
**[71]**	Yes	Yes	Yes	Yes	No	No	No	No
**[48]**	No	Yes	No	No	No	Yes	No	No
**[81]**	No	No	No	Yes	Yes	No	Yes	No
**[28]**	Yes	Yes	No	No	No	Yes	No	No
**[35]**	Yes	Yes	Yes	No	No	Yes	Yes	Yes
**[83]**	No	Yes	No	Yes	Yes	Yes	Yes	No
**[60]**	Yes	Yes	No	Yes	No	Yes	No	No
**[51]**	Yes	No	Yes	Yes	Yes	Yes	No	No
**[33]**	No	No	Yes	Yes	Yes	Yes	No	No
**[40]**	No	Yes	Yes	No	No	Yes	No	No
**[50]**	No	Yes	No	Yes	No	Yes	No	Yes
**[59]**	No	Yes	No	Yes	Yes	Yes	No	No

## Appendix A

**Table A1:** Mathematical comparison of repeated game approach.

Reference	[73]	[34]	[74]	[75]
Set of finite agents	N=[1,2,3,…,n]	$T_{i},i=1,2,\ldots ,N$	$T=(t_{i},\ldots ,t_{y})$	$\begin{array}{l} P=[x_{v}(1),\\ x_{v}(2),\ldots ,x_{v}(T)] \end{array}$
Set of neighbor agents	$N_{i}=[j|M_{ij}=1]$	P=[P(i,j)]	$S_{i}^{\prime }=n(S_{i},a_{i})$	$S=X\times Y\times Z\times 2^{N}$
Agent set of action	$A_{i}=[\emptyset \sigma_{i},1,2,\ldots ,k_{i},\infty ]$	$V\beta ={\left[v\beta (S)\right]}_{s}\in S$	$MDP=(S_{i},A_{i},T_{i},R_{i})$	$C(x_{v},x_{n})=f(D(x_{v},x_{n}))$
Payoff	$\begin{array}{l} u_{i}^{t}(\theta_{i},a_{i}^{t},a^{t}{\_}_{i},S)=-c\\ u_{i}^{t}(\theta_{i},a_{i}^{t},a^{t}{\_}_{i},S)=0 \end{array}$	$u_{i}(S)=u_{i}=(S_{i},S_{-i})$	$u_{i}=x_{i}(s_{1},a_{i},s_{i}^{\prime })R_{i}$	$R(x_{v})=\sum_{n\in N}P(x_{v},x_{n})L(Y_{n})$
Utility function	$\begin{array}{l} u_{i}(\theta_{i},\sigma_{i},\sigma_{-i},S)=\\ \sum_{t>0}u_{i}^{t}(\theta_{i},a_{i}^{t},a_{-i},S) \end{array}$	$\begin{array}{l} R(S^{\left(k\right)},m,l)=\\ \left[1ifm/\in L_{k}\&m\neq l\right] \end{array}$	$\begin{array}{l} x_{i}(s_{1},a_{i},s_{i}^{\prime })=\\ \sum_{t\in x}\varphi (t,\pi )\theta (t_{1},s_{1},a_{i},s_{i}^{\prime }) \end{array}$	$\begin{array}{l} R(S,S^{\prime })=\\ \sum_{n\in F}L(Y_{n})-1c(x_{s},x_{s}^{\prime }) \end{array}$
History/Set of history	$H^{t}=U_{t\geq 0}H_{\infty }^{t}$ $\begin{array}{l} H^{t}=U_{t\geq 0}H_{\infty }^{t} \end{array}$	$\sum_{i=1}^{n}\vec{T}=T$	$M=\sum_{t}|S_{i}|A_{i}|$	$Z=L(Y_{n},Y_{m})$
Set of action profile	$\begin{array}{l} u_{i}=\min\max u_{i}(\theta_{i},a_{i},a_{-i})\\ a_{i}\in A_{i},a_{-i}\in A_{i} \end{array}$	$S={\left[S_{i}\right]}_{i=1}^{n}$ $S={\left[S_{i}\right]}_{i=1}^{n}$	$w_{i}(s_{i},a_{i})\geq 0\forall s_{i},a_{i}$	P*=argmaxR(P)−1
Payoff vector	$\begin{array}{l} V:=[x=(x_{1},\ldots ,x_{n})\in F:\\ x_{1}\geq u_{1}\forall_{i}\in N] \end{array}$	vβ=val[R(S,Vβ)]	$\begin{array}{l} x_{i}(s_{1},a_{i},s_{i}^{\prime })=w_{i}(s_{i},a_{i})\\ T_{i}(s_{1},a_{i},s_{i}^{\prime })\forall s_{1},a_{i},s_{i}^{\prime } \end{array}$	$c(x_{v},x_{n})=p(x_{v},x_{n})$

## Appendix B

**Table A2:** Mathematical Comparison of Dynamic Bayesian games.

Reference	[32]					[76]				
No of prayers/No of actions	2/2					2/2				
Type of action	same					different				
SPC & RPC	$\begin{array}{l} u_{1}=(\sigma_{1},\sigma_{2},\theta )=\\ \sum_{a_{1}}\sum_{a_{2}}\sigma_{1}(a_{1}|\Theta )\sigma_{2}(a_{2}|a_{1})u_{1}(a_{1},a_{2},\Theta ) \end{array}$ and $\begin{array}{l} u_{2}=(\sigma_{1},\sigma_{2},\theta )=\\ \sum_{\Theta }P(\Theta )\sum_{a_{1}}\sum_{a_{2}}\sigma_{1}(a_{1}|\Theta )\sigma_{2}(a_{2}|a_{1})u_{2}(a_{1},a_{2},\Theta ) \end{array}$					$\begin{array}{l} u_{1}=(\sigma_{1},\sigma_{2},\theta )=\\ \sum_{a_{1}}\sum_{a_{2}}\sigma_{1}(a_{1}|\Theta )\sigma_{2}(a_{2}|a_{1})u_{1}(a_{1},a_{2},\Theta ) \end{array}$ and $\begin{array}{l} u_{2}=(\sigma_{1},\sigma_{2},\theta )=\\ \sum_{\Theta }P(\Theta )\sum_{a_{1}}\sum_{a_{2}}\sigma_{1}(a_{1}|\Theta )\sigma_{2}(a_{2}|a_{1})u_{2}(a_{1},a_{2},\Theta ) \end{array}$				
Strategy Profile of PBE of S & R	$P_{s}:\forall \Theta ,u_{1}(\sigma_{1}^{*},\sigma_{2}^{*},\theta )\geq u_{1}(\sigma_{1},\sigma_{2}^{*},\theta )$ and $P_{R}:\forall \Theta ,u_{2}(\sigma_{1}^{*},\sigma_{2}^{*},\mu^{*})\geq u_{2}(\sigma_{1}^{*},\sigma_{2},\mu^{*})$					$P_{s}:\forall \Theta ,u_{1}(\sigma_{1}^{*},\sigma_{2}^{*},\theta )\geq u_{1}(\sigma_{1},\sigma_{2}^{*},\theta )$ and $P_{R}:\forall \Theta ,u_{2}(\sigma_{1}^{*},\sigma_{2}^{*},\mu^{*})\geq u_{2}(\sigma_{1}^{*},\sigma_{2},\mu^{*})$				
Belief of their type Θ & message m	$P_{B}:\mu^{*}(\Theta |a_{1})=\frac{P_{D}(\theta )\sigma^{*}(a_{1}|\Theta )}{\sum_{\theta \in \Theta }P(\theta^{\prime })\sigma_{1}^{*}(a_{1}|\Theta^{\prime })}$ and $P(\theta |m)=\frac{P(\theta^{\prime })\sigma (m|\theta )}{P(\theta )\sigma (m|\theta )}$ $P(\theta |m)=\frac{P(\theta^{\prime })\sigma (m|\theta )}{P(\theta )\sigma (m|\theta )}$					$P_{B}:\mu^{*}(\Theta |a_{1})=\frac{P_{D}(\theta )\sigma^{*}(a_{1}|\Theta )}{\sum_{\theta \in \Theta }P(\theta^{\prime })\sigma_{1}^{*}(a_{1}|\Theta^{\prime })}$ and $P(\theta |m)=\frac{P(\theta^{\prime })\sigma (m|\theta )}{P(\theta )\sigma (m|\theta )}$				
Best Response of S & R	$\sum_{\alpha \in A}\rho (\alpha |m)\mu (m,\alpha ,\theta )$ and $V(\alpha ,\sigma )\equiv \sum_{m\in M}\sum_{\theta \in \Theta }\rho (\theta )\sigma (m|\theta )v(m,\alpha (m),\theta )$					$\sum_{\alpha \in A}\rho (\alpha |m)\mu (m,\alpha ,\theta )$ and $V(\alpha ,\sigma )\equiv \sum_{m\in M}\sum_{\theta \in \Theta }\rho (\theta )\sigma (m|\theta )v(m,\alpha (m),\theta )$				
Payoff Matrix and Nash	Senders message	Nodes type		Receivers action		S message	Nodes type		Receivers action	
		Normal	malicious	cooperate	Decline		regular	malicious	cooperate	Decline
		PS	PS	1, P	−1, P − 1		PS	PS	1, P	−1, P − 1
		PS	SM	P, P	P, 0		PS	SM	P, P	P, 0
		SM	PS	1 − P, 0	P − 1, P − 1		SM	PS	1 − P, 0	P − 1, P − 1
		SM	SM	0, 0	−1, P-1		SM	SM	0, 0	−1, P − 1
	(PS PS,C) & (SM SM,D)					(PS PS,C) & (SM SM,D)				

## Appendix C

**Table A3:** Mathematical Comparison of Dynamic Bayesian games continuation.

Reference	No. of Players	Type of Action	Payoff Formulation	Player Belief	Nash
[34]	2	Same	$X^{*}:={\left(x_{i}\right)}_{i}^{n}=1$	$\mu_{0}=\frac{(1+\beta )\omega +c_{m}}{(2\alpha +\beta -1)\omega }$	$\mu_{i}(S_{i}^{*},S_{-i}^{*})\geq \mu_{i}(S_{i}^{*},S_{-i}^{*})\forall s_{i}^{*}\in S_{i})$
[48]	2	Different	$f_{ij}=\frac{r_{ij}+1}{2}\times \frac{d_{ij}}{\max d_{i}}$ $\mu (T(n,\delta )=\sum_{t=1}u_{t}(T)\delta^{t}$	$f_{ij}=\frac{r_{ij}+1}{2}\times \frac{d_{ij}}{\max d_{i}}$	$C_{true}C_{false}(DD)$
[78]	2	Same	$u_{x}=u(h_{j}^{i}(t_{k+1}),a_{i},a_{j},\theta_{i},\theta_{j})$	$\mu_{i}^{j}(t_{k+1})=\rho (\theta_{i}^{j}(t_{k})h_{i}^{j}(t_{k}),f_{i}^{j}(t_{k}))$	…
[81]	2	Same	$u_{i}(T)=(G_{p}-C_{p})P(1-\theta_{j})+(G_{p}-C_{p})(1-P)+(C_{p})P\theta_{j}$	$\mu_{i}(t_{j}(h_{j}^{t},a_{j}(t)))=\frac{\mu (t_{j}|(h_{j}^{t}))P(a_{j})(t)(h_{j}^{t},t_{j})}{\sum_{t\in T_{j}}\mu_{i}(t_{j}|(h_{j}^{t}))P(a_{j})(t)(h_{j}^{t},t_{j})}$	$\left(\sigma_{j},\sigma_{i},C)(\sigma_{j},\sigma_{i},O\right)$
[83]	2	Same	$F(T,{}_{T})-\sum_{t=1}^{T}Pt,\sum_{t=1}^{T}Pt$	$P(U>pU>p*)=P(U>p)\frac{1}{P(U>p*)}$	$S_{C}^{*}(WB)S_{C}^{*}(FT)$
[77]	2	Different	$PK_{j}=H_{1}(UID_{j}||\mathrm{Pr}i_{j})\in G$	$ê(\sigma_{j}P)=ê(H_{2}(m)SK_{j}P)=ê(H_{2}(m)SPK_{j}P)$	$\left(PK_{j},SK_{j},\mathrm{Pr}i_{j})(SK_{j},U_{j}\right)$
[79]	2	Different	$G_{S}(W_{S})-G_{S}(W_{S})=(G_{ch}+G_{m})+(f_{a}p_{att}^{a}p_{s}^{a}+f_{b}p_{att}^{b}p_{s}^{b})-C_{WD})$	$P_{Wa}^{S}=-\frac{C_{a}+G_{ch},P_{Wa}^{S}}{G_{ch}+G_{ca}}=P_{s}^{a}=\frac{C_{a}+G_{ch}}{G_{ch}+G_{ca}}$	…
[80]	2	Same	$\phi =(G_{C}-C_{C})/((G_{C}+G_{A})\theta )$	$u=\frac{12\alpha \beta }{{\left(\alpha +\beta \right)}^{2}(\alpha +\beta +1)}$	$\begin{array}{l} \left(\sigma_{j}\sigma_{i},A)or(\sigma_{j}\sigma_{i}C\right)\\ \left(\sigma_{j}\sigma_{i},\phi )or(\sigma_{j}\sigma_{i}\rho \right) \end{array}$

## Appendix D

**Table A4:** Mathematical Comparison of Evolutionary Games.

Author(s) & Year	[51,83,84]	[71]		[50,85]	[86,87]
Population behavior	$S=(a_{1},a_{2},\ldots ,a_{n})$	$S=(S_{1},S_{2},\ldots ,S_{k})$	$S=(S_{1},S_{2},\ldots ,S_{k})$	$r=(r_{1},r_{2},r_{3},\ldots ,r_{n})$	$\omega =(\omega_{1},\omega_{2},\ldots ,\omega_{n})$
Individual pure strategy	$\begin{array}{l} \theta (t)=\\ \left(\theta_{1}(t),\theta_{2}(t),\ldots ,\theta_{n}(t)\right) \end{array}$	$X=(x_{1},x_{2},\ldots ,x_{k})$	$X=(x_{1},x_{2},\ldots ,x_{k})$	$X=(x_{1},x_{2},x_{3},\ldots ,x_{n})$	$a=(a_{1},a_{2},\ldots ,a_{n})$
Population mixed strategy	$\theta_{i}(t)=\frac{\phi_{i}(t)}{\sum_{i}\phi_{i}(t)}$	$u(X,X)=\sum_{i\in S}X_{j}u(i,X)$	$u(X,X)=\sum_{j\in S}X_{j}u(i,X)$	$\begin{array}{l} P(X_{ti}|X_{11})=\\ \sum \lambda p(X_{ti}|X_{11})P \end{array}$	$\begin{array}{l} g_{i}(\alpha )=\\ \sum_{\omega }p(\omega |\alpha )u_{i}(\alpha_{i},\omega_{i}) \end{array}$
Individual payoff	$\mu (a_{i},\theta (t)\sum_{i}\theta_{j}(t)\mu (a_{i},a_{j})$	$\begin{array}{l} F(X_{i})=\\ X_{i}[u(i,X)-u(X,X)] \end{array}$	$\begin{array}{l} F(X_{i})=\\ X_{i}\sum_{j\in S}X_{j}[p_{j}^{i}(X)p_{i}^{j}(X)] \end{array}$	$\begin{array}{l} U(q\in p+(1\in q))>\\ U(q\in p+(1\in q) \end{array}$	$U_{i}(S^{C},S^{C})\geq U_{i}(S^{\alpha },S^{C})$
Population payoff	$\begin{array}{l} \mu (\theta (t),\theta (t))\\ \sum_{i}\theta_{i}(t)\mu (a_{i},\theta (t)) \end{array}$	$\begin{array}{l} F(X_{i})=\\ X_{i}\sum_{i\in S}X_{i}[p_{j}^{i}(X)p_{i}^{j}(X)] \end{array}$	$\begin{array}{l} F(X_{i})=\\ X_{i}[u(i,X)-u(X,X)] \end{array}$	$\begin{array}{l} u_{i}(\alpha )=\\ \sum_{\theta \in \Theta }u_{i}(\alpha_{i},\alpha_{-i},\theta_{i})\mathrm{Pr}ob\\ \geq U_{i}(\theta_{i}|\alpha_{-i}) \end{array}$	$\begin{array}{l} X*=\\ \left[u(S^{*},S^{C})-u(S^{C},S^{C})\right] \end{array}$
Payoff Metrix	$\begin{array}{l} \omega_{T}+\omega_{C}+\alpha T+2\beta +\omega_{T}+\\ \alpha T-\beta -\gamma \\ \omega_{M}+\omega_{C}-\beta \omega_{M} \end{array}$	$\begin{array}{l} G_{T}+G_{C}+\alpha T-2C_{2}\alpha T-C_{2}-L\\ G_{T}+\alpha T-2C_{2}-LG_{C}+\alpha T-C_{2} \end{array}$	$\begin{array}{l} G_{T}+G_{C}+\alpha T-2C_{2}\alpha T-C_{2}-L\\ G_{T}+\alpha T-2C_{2}-LG_{C}+\alpha T-C_{2} \end{array}$	$\begin{array}{l} b(1-p_{e})-c\\ b(1-p_{e})0 \end{array}$	$\begin{array}{l} u(S^{*},S^{*})u(S^{*},S^{C})\\ u(S^{C},S^{*})u(S^{C},S^{C}) \end{array}$
Trust evaluation	$\delta_{3}^{*}=\frac{(\omega_{M}+\beta +\gamma +\omega_{T}-\alpha T}{\gamma }$	$\begin{array}{l} F(X)=X(1-X)\\ {}[\omega^{2}G_{T}+\alpha T-(\omega -\omega^{2})C_{1}\\ -\omega^{2}C_{2}] \end{array}$		…	…

## Appendix E

**Table A5:** Mathematical Comparison of Bargaining Games.

Papers	[89]	[90]
**Potential Buyer**	$n_{0}$	$U_{i}(b_{i})$
**Group of Potential sellers**	$N=(n_{1},n_{2},\ldots ,n_{N})$	$(U_{1}(b_{1}),\ldots ,U_{n}(b_{n}))\in U$
**Buyer request**	$n_{0}↔NN_{S}\in \mathbb{N}(m)$	$(U_{1}(b_{1}),\ldots ,U_{n}(b_{n}))\in U$
**Bidding price &**	$b_{0}\forall n_{0}\;if\;r_{0}\geq b_{0}$	$F(U,d)=[I\in BS]I=\sum_{i=1}\beta_{i}v_{i},\sum_{i=1}\beta_{i}=1,\beta_{i}\geq \forall_{i}]$
**Sealed offer**	$b_{0}\&S_{i}(1<i<N)\;for\;(n_{0}\&NN_{S})$	$U_{i}(b_{i})=b_{i}\geq R_{0i},R_{0i}>0,k\geq 0$
**Set of coalition**	$C=[C\in 2^{N}\sum S_{i}\leq b_{0}\&|C|\geq \min CN]$	…
**Bargaining @ agreed price**	$P=\varepsilon Xb_{0}+(1-\varepsilon )X\sum S_{i}$	$BS=(S(S_{i},\ldots ,S_{n})=B\;\max-\sum R_{0i},S_{i}>0,\forall_{i}$

## Appendix F

**Table A6:** Mathematical Comparison of Coalition Games.

Reference	[91]	[92]	[24]	[72]
Players	N=[1,2,…,N]	$T=(t_{1},t_{2},\ldots ,t_{m})$	I=[1,2,…,M]	X=[1,2,…,X]
Coalition	S⊆N	$S_{1},S_{2}\subset N$	$S_{1}\in S_{i\forall }\forall \subset S_{i}\in I$	Z⊂X∈XZ
Transferable utility	$\sum_{i\in N}X_{i}=v(N)$	$\sum_{i\in S}P_{i}h_{i}=\gamma_{i}$	$\sum_{i\in S}P_{i}^{k}=P^{i}$	$\sum_{i\in Y}X_{i}=V(X)$
In characteristics form	$v(s_{1},[s_{2},s_{3}])=v(s_{1},[s_{4}])=v(s_{1})$	$v(s_{1}\vee s_{2})\geq v(s_{1})+v(s_{2})$	$S_{X}^{*}(a_{-i},a_{i})Q_{i}^{s}\geq (X,[a_{-i},a_{i}])$	$v(Z_{1},[Z_{2},Z_{3}])=v(Z_{1},[Z_{4}])=v(Z_{1})$
In partition form	$v(s_{1},[s_{2},s_{3}])\neq v(s_{1},[s_{4}])$	$v(s_{1}\vee s_{2})\neq v(s_{1})+v(s_{2})$	$S_{X}^{*}(a_{-i},a_{i})Q_{i}^{s}\neq (X,[a_{-i},a_{i}])$	$v(Z_{1},[Z_{2},Z_{3}])\neq v(Z_{1},[Z_{4}])$
In graph form	$v(G_{S}^{1})\neq v(G_{S}^{2})$	$G\sum_{i\in S}P_{i}=p_{t}xg_{t}$	$\sum S^{*}\in S_{i},\forall S_{i}\neq S_{i}^{*}$	$v(C_{S}^{1})\neq v(C_{S}^{2})$
Payoff vector	$\begin{array}{l} \left[X:\sum X_{i}=v(N)\&\sum X_{i}\geq v(S)\right]\\ \forall S\subset N \end{array}$	$\begin{array}{l} v(S)=-\sum_{i\in s}p_{i}-p_{0}\\ if\;\gamma_{s},R,d>\gamma \end{array}$	$\begin{array}{l} u_{i}(P,A^{i})=\\ \sum \beta_{i},R_{i}(P,A^{i}) \end{array}$	$\begin{array}{l} \left[Y:\sum X_{i}=v(X)\&\sum X_{i}\geq v(S)\right]\\ \forall S\subset N \end{array}$
